# Comparison of STR-Based Genetic Diversity Between Young and Elderly Populations and Its Association with Aging-Related Pathways

**DOI:** 10.3390/genes17080897

**Published:** 2026-07-30

**Authors:** Kursat Ozdilli, Yeliz Ogret, Suleyman Rustu Oguz, Cigdem Kekik Cinar, Fatma Oguz

**Affiliations:** 1 Department of Medical Biology, Faculty of Medicine, Istanbul Medipol University, 34810 Istanbul, Türkiye; kozdilli@medipol.edu.tr; 2 Tissue Typing Laboratory, Department of Medical Biology, Faculty of Medicine, Istanbul University, 34093 Istanbul, Türkiye; cigdem.kekik@istanbul.edu.tr (C.K.C.); oguzsf@istanbul.edu.tr (F.O.); 3 Department of Medical Biology and Genetic, Faculty of Medicine, Demiroglu Bilim University, 34394 Istanbul, Turkey; rustu.oguz@florence.com.tr

**Keywords:** short tandem repeat (STR), aging, genetic diversity, KEGG pathway analysis, heterozygosity

## Abstract

**Background/Objectives**: Aging is characterized by genomic instability, reduced biological diversity, and clonal dominance across multiple biological systems. While short tandem repeats (STRs) are traditionally considered neutral genetic markers, emerging evidence raises the possibility that variation in these regions may be examined in relation to broader genomic processes associated with aging. **Methods**: STR profiles from 400 individuals were analyzed, including 275 young participants aged 21–43 years and 125 elderly participants aged ≥65 years. Genetic diversity was assessed using parameters such as expected heterozygosity (He), individual heterozygosity ratio, and allelic dominance. Additionally, STR loci were mapped to the hg38 reference genome (±100 kb), and nearby genes were annotated with KEGG pathway information to provide exploratory biological context for the investigated loci. **Results**: No statistically significant differences were observed in heterozygosity or allelic dominance between the young and elderly groups. However, a slight trend toward decreased heterozygosity and increased allelic dominance was noted in the elderly population. Genomic mapping indicated that some STR loci are located in proximity to genes annotated in aging-related pathways; however, this finding is based on genomic proximity and should be interpreted as exploratory. **Conclusions**: The primary analyses did not demonstrate statistically significant differences in STR-based genetic diversity between the young and elderly groups. The observed trends and genomic proximity findings should therefore be considered exploratory and hypothesis-generating. Validation in larger, independent prospective cohorts and integration with functional genomic data are required before any biological significance can be inferred.

## 1. Introduction

Genomic variations form the basis of interindividual differences, and a significant portion of this variation is accounted for by highly polymorphic DNA regions such as short tandem repeats (STRs). STRs are genetic markers composed of sequential repeats of short DNA motifs that exhibit extensive allelic diversity across populations due to high mutation rates. They account for approximately 3–5% of the entire genome and consist of short sequence repeats ranging from 1 to 6 base pairs [1]. Due to their repetitive structure, STRs are prone to misalignment errors during DNA replication, which leads to increased mutation rates in these regions. Thanks to these characteristics, STRs have been widely used in forensic genetic and population genetic studies for many years [2,3].

Although STR loci are generally considered neutral markers, it has been suggested in recent years that the changes observed in these regions may reflect not only random variations but also broader genomic processes. In particular, the concept of microsatellite instability (MSI) describes length changes and mutation accumulation in such repeat sequences and is recognized as an important indicator of genomic instability [4]. MSI primarily arises from defects in the DNA mismatch repair (MMR) system, resulting in the accumulation of mutations in repetitive regions due to the inability to correct replication errors [5,6,7]. However, the present study does not assess somatic microsatellite instability but rather focuses on interindividual germline variation.

The relationship between the aging process and genomic instability has long been a subject of research. In current biological models, “genomic instability” is recognized as one of the key determinants of aging. It has been demonstrated that, with advancing age, DNA damage accumulates, the efficiency of DNA repair mechanisms declines, and the burden of somatic mutations increases. In this context, accumulating evidence indicates that microsatellite instability (MSI) becomes more frequent in somatic tissues during aging [8]. Studies using peripheral blood and other somatic cell types have reported age-associated increases in microsatellite mutations, which are thought to reflect declining genomic maintenance and DNA repair capacity [9]. However, these observations relate to somatic microsatellite instability rather than inherited germline STR variation, which is the focus of the present study. Although these biological processes are distinct, evidence of age-related genomic instability provides a rationale for investigating whether germline STR diversity differs between age groups at the population level. However, aging is characterized not only by the accumulation of mutations but also by a decline in biological diversity and the dominance of specific clones. In particular, repertoire contraction and clonal expansion observed in the immune system represent well-established hallmarks of aging. These processes lead to reduced variability and increasingly skewed distributions at the system level. This raises the hypothesis that STR-based parameters may be explored in relation to broader age-associated patterns of genomic variation [9,10].

In this context, parameters such as the level of heterozygosity observed at STR loci, allele frequency distribution, and allelic dominance can be evaluated not only as measures of population genetics but also as indirect indicators of genomic remodeling associated with aging [8,9,11]. In particular, patterns such as a decrease in heterozygosity and the dominance of certain alleles in elderly populations may reflect a situation where genomic diversity is reduced and variation shifts toward a more restricted structure.

Furthermore, given the locations of STR loci within the genome, it is worth noting that these regions may be located in genomic proximity to genes annotated in biological pathways associated with aging. Processes such as cellular senescence, inflammation, DNA repair, and metabolic regulation are among the fundamental components of aging [9,10], and it is known that genes regulating these processes are clustered in specific genomic regions [12,13]. Therefore, analyzing the genomic context of STR loci may contribute to interpreting the biological significance of the observed genetic patterns.

Although STRs have been extensively studied in forensic and population genetics, evidence regarding their stability and variation across the human lifespan remains limited. Recent population-scale sequencing studies have enabled direct characterization of STR variation, mutation rates, and repeat instability in large human cohorts. Analyses of the 1000 Genomes Project and other deeply sequenced populations have demonstrated extensive genome-wide STR polymorphism, whereas studies using UK Biobank data have identified germline mutation patterns, phenotypic associations, and age-related somatic instability at selected tandem-repeat loci. However, these studies have generally focused on genome-wide repeat variation, mutation mechanisms, or disease-associated repeats rather than on conventional forensic STR heterozygosity across age groups. Therefore, the relationship between aging and population-level variation in forensic STR alleles remains insufficiently characterized [14,15].

In this study, STR-based genetic diversity was compared between young and elderly populations; age-related changes were evaluated based on parameters such as heterozygosity, allele frequency, and allelic dominance. Additionally, by analyzing the genomic locations of STR loci, the study investigated how these changes align conceptually with biological pathways associated with aging. It should be noted that the present study does not assess somatic microsatellite instability (MSI), but rather evaluates interindividual germline variation. Therefore, the findings should not be interpreted as reflecting mutation accumulation within individuals over time.

## 2. Materials and Methods

### 2.1. Study Population

In this study, the elderly group was operationally defined as individuals aged ≥65 years [16]. The study included two age-defined groups from the same population. The young group consisted of healthy individuals without chronic diseases or inflammatory conditions. Individuals with active malignancies (including hematological malignancies) and autoimmune diseases were excluded from the elderly group. The young group consisted of 275 individuals aged 21–43 years (mean age: 31.2 ± 6.3), whereas the elderly group consisted of 125 individuals aged 65–91 years (mean age: 74.66 ± 7.81). All individuals were selected to represent the same population structure, and no known kinship relationships existed among the participants. The demographic information (age) of the individuals was used in the analyses. All participants were recruited from the same geographical region (Istanbul, Türkiye).

### 2.2. STR Genotyping

Peripheral whole blood samples were collected in EDTA-containing tubes from all participants. Genomic DNA was extracted using the EZ1 Advanced XL automated extraction system (Qiagen, Venlo, The Netherlands) according to the manufacturer’s protocol. DNA concentration and purity were measured using a NanoDrop 2000/2000C spectrophotometer (Thermo Fisher Scientific, Waltham, MA, USA). STR loci were amplified using the AmpFlSTR Identifiler PCR Amplification Kit (Applied Biosystems, USA) according to the manufacturer’s protocol. This multiplex system targets the following loci: D8S1179, D21S11, D7S820, CSF1PO, D3S1358, TH01, D13S317, D16S539, D2S1338, D19S433, vWA, TPOX, D18S51, D5S818, and FGA.

PCR amplification was performed following the standard cycling conditions recommended by the manufacturer. Amplified products were separated by capillary electrophoresis using an ABI Prism 3130 XL Genetic Analyzer (Applied Biosystems, USA), and allele calling was carried out using GeneMapper v5.0 software. Genotype data were obtained for all individuals and used for subsequent analyses.

### 2.3. Genetic Diversity Analyses

Various parameters were calculated to assess genetic diversity among populations. Expected heterozygosity (He): Calculated based on allele frequencies at the locus level. Individual heterozygosity ratio: Determined as the ratio of the number of heterozygous loci to the total number of loci in each individual. Maximum allele frequency (allelic dominance): Allelic dominance was assessed by calculating the frequency of the most common allele at each locus. Allele frequency calculations and basic population genetic analyses were performed using Arlequin (v3.5) software [17]. Statistical analyses were performed using the Mann–Whitney U test to compare genetic diversity parameters between groups. A *p*-value < 0.05 was considered statistically significant. Additional subgroup analyses were performed to evaluate whether age-related trends became more apparent in advanced age ranges. The elderly cohort was stratified into three age subgroups (65–74, 75–84, and ≥85 years). In addition, an extreme-age comparison was performed between younger individuals aged ≤30 years and elderly individuals aged ≥85 years. Statistical comparisons among multiple subgroups were conducted using the Kruskal–Wallis test, followed by pairwise Mann–Whitney U tests where appropriate.

Hardy–Weinberg equilibrium (HWE) was evaluated separately in the young and elderly groups for all 15 autosomal STR loci using the Monte Carlo exact test implemented in the HWE function of the R package *gap*, with 100,000 Monte Carlo replicates. To account for multiple testing across the 15 loci, Bonferroni correction was applied separately within each study group, resulting in a corrected significance threshold of *p* < 0.0033.

During the preparation of this manuscript, ChatGPT (OpenAI) was used only to assist in the graphical layout and visual refinement of Figure 5 and for language editing. ChatGPT was not used to generate, analyze, or interpret the scientific data or study results. All numerical values, pathway annotations, labels, and scientific content presented in Figure 5 were derived from the original analyses and were independently verified by the authors against the original study data before submission. The authors take full responsibility for the accuracy and integrity of all content in this manuscript.

### 2.4. Exploratory Mapping of STR-Adjacent Genes to KEGG Pathways

To explore the potential biological context of STR loci, genomic coordinates of each STR marker were obtained based on the hg38 reference genome. A ±100 kb window was selected to identify genes located in close genomic proximity to each STR locus, as regulatory elements such as enhancers may act over this range, and this distance is commonly used in proximity-based genomic analyses. Identified genes were subsequently mapped to KEGG pathway annotations to provide biological context for the investigated loci. The pathway analysis was intended as an exploratory functional annotation based on genomic proximity and was not designed as a comprehensive statistical enrichment analysis using a predefined genomic background. Consequently, the identified pathway associations should be interpreted as hypothesis-generating and do not imply direct functional, causal, or regulatory relationships. Given the exploratory nature of the pathway annotation, no formal enrichment hypothesis was prespecified. No a priori sample-size or statistical power calculation was performed for the group comparisons.

## 3. Results

### 3.1. Age-Related Differences in STR-Based Genetic Diversity

**Figure 1:**
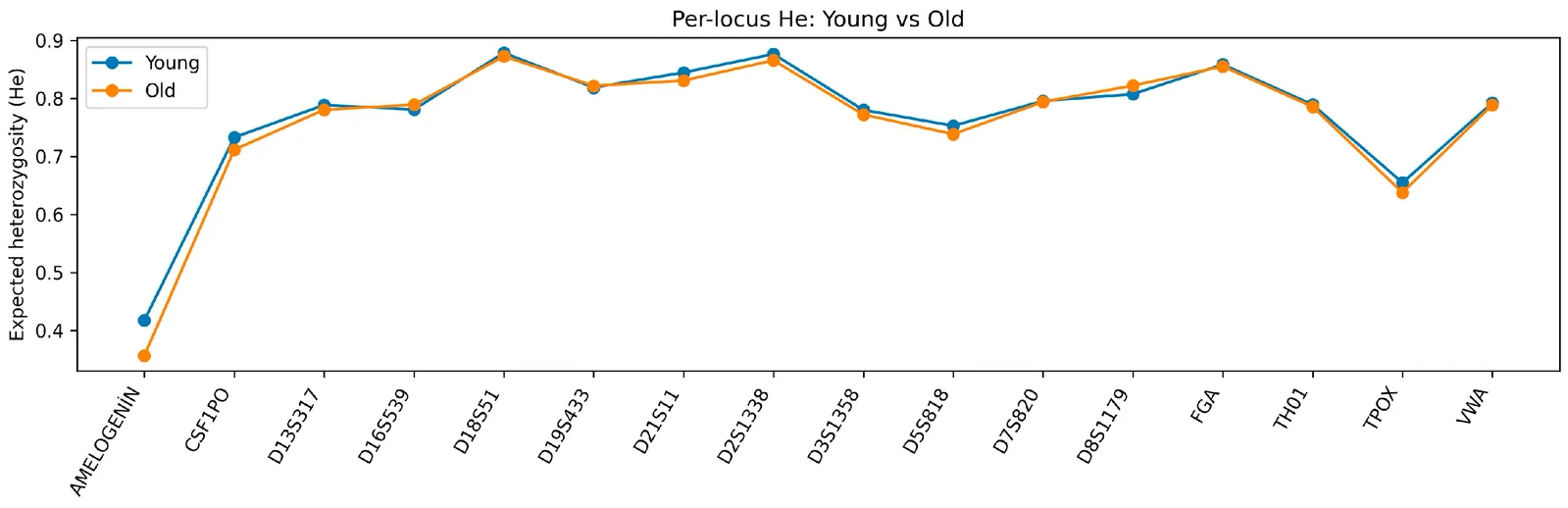
Per-locus expected heterozygosity (He) comparison between young and elderly groups. No statistically significant difference was observed between the groups (Mann–Whitney U test, *p* > 0.05).

Locus-specific analyses of expected heterozygosity (He) showed similar distributions between the young and elderly groups (Figure 1). Although a slight decrease in He values was observed in the elderly group across several loci, this difference did not reach statistical significance (Mann–Whitney U test, *p* > 0.05).

**Figure 2:**
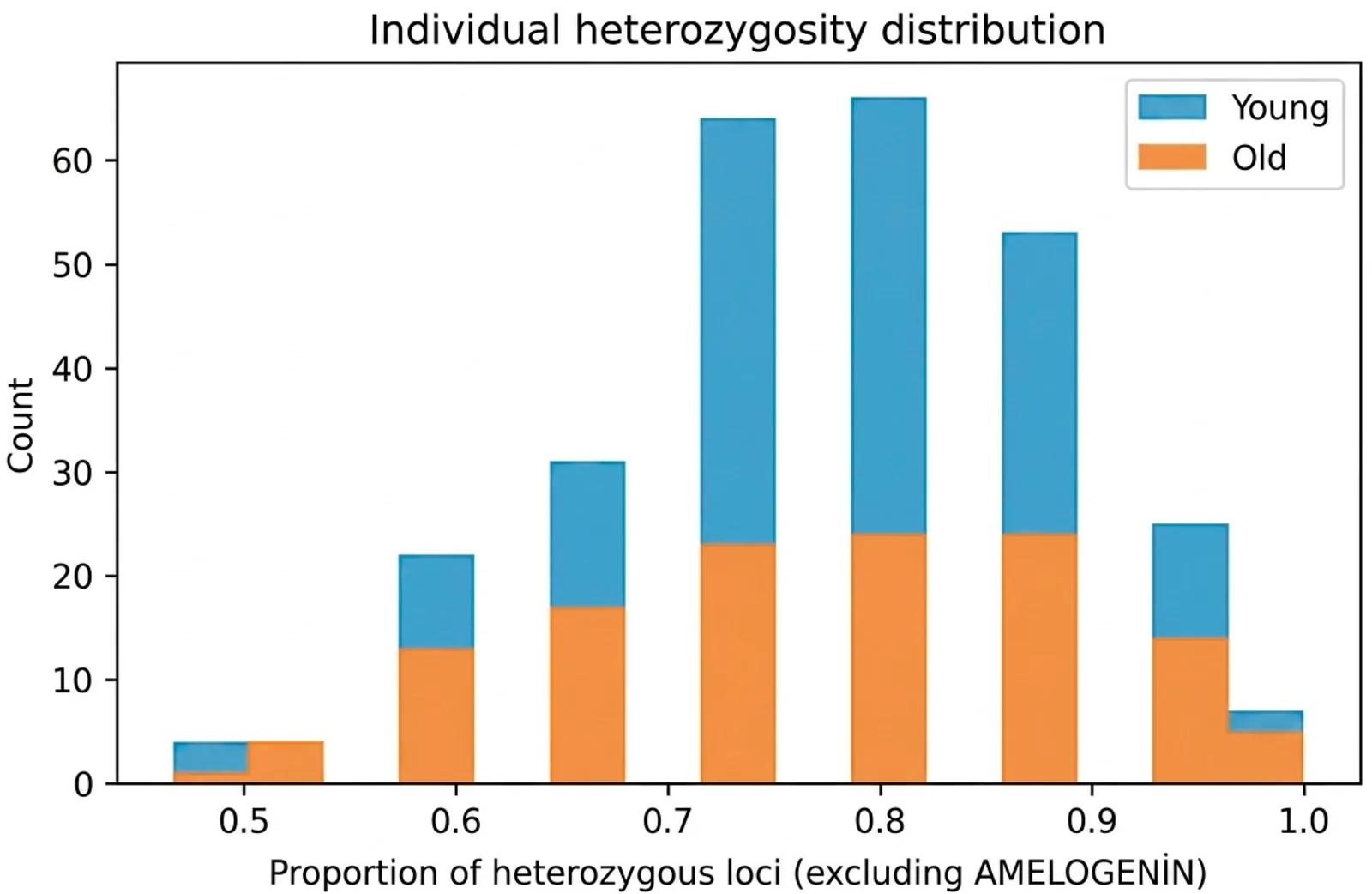
Distribution of individual heterozygosity ratios in the young (n = 275) and elderly (n = 125) groups. Additional age-subgroup analyses included individuals aged ≤30 years (n = 124), 65–74 years (n = 75), 75–84 years (n = 31), and ≥85 years (n = 19).

At the individual level, the distribution of heterozygosity ratios was similar between the two groups (Figure 2). A minor shift toward lower heterozygosity values in the elderly group was observed; however, this difference did not reach statistical significance (Mann–Whitney U test, *p* > 0.05). To further investigate whether age-associated trends became more apparent in advanced age ranges, additional subgroup analyses were performed within the elderly cohort (65–74, 75–84, and ≥85 years). The elderly cohort comprised 75 individuals aged 65–74 years, 31 individuals aged 75–84 years, and 19 individuals aged ≥85 years. For the extreme-age comparison, 124 individuals aged ≤30 years were compared with 19 individuals aged ≥85 years. A statistically significant difference in individual heterozygosity ratios was observed among these age subgroups (Kruskal–Wallis test, *p* = 0.040). The ≥85 years subgroup showed the lowest heterozygosity levels compared with the other elderly age groups. Furthermore, an extreme-age comparison between younger individuals aged ≤30 years and elderly individuals aged ≥85 years demonstrated significantly lower heterozygosity in the ≥85 years group (Mann–Whitney U test, *p* = 0.023).

When evaluated together, these findings do not indicate a statistically significant difference in STR-based genetic diversity between young and elderly populations, although a slight trend toward reduced diversity in the elderly group was observed.

### 3.2. An Increased Pattern of Allelic Dominance in the Elderly Group

**Figure 3:**
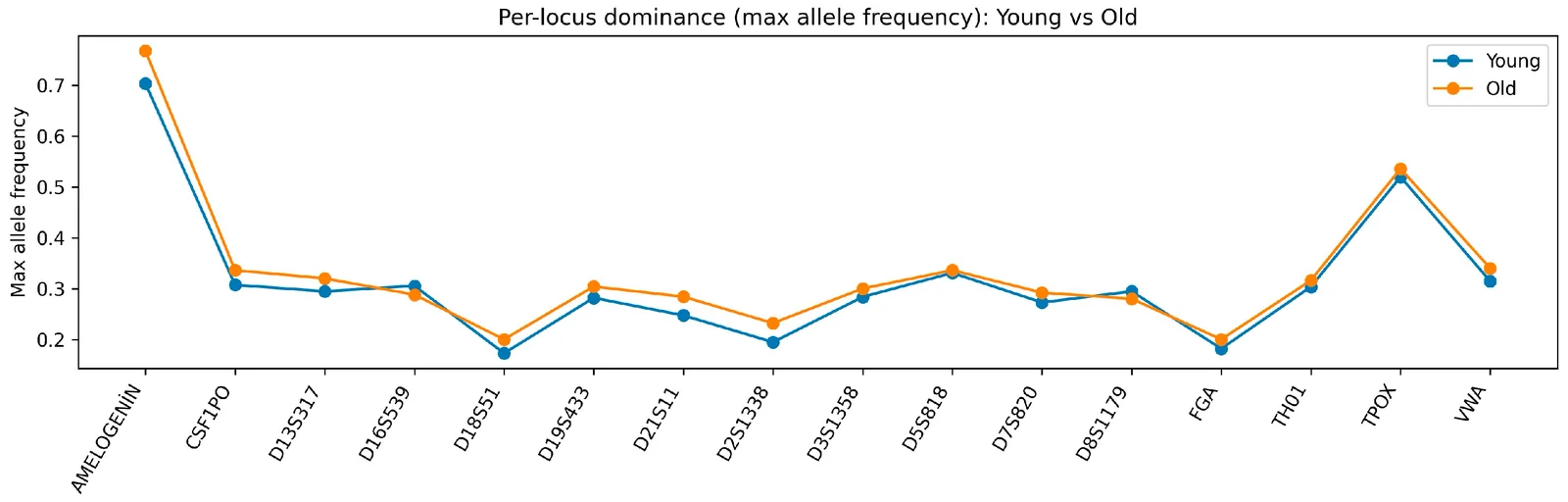
Per-locus maximum allele frequency comparison between the young and elderly groups. Maximum allele frequency was used as a measure of allelic dominance. No statistically significant difference was observed between the groups (Mann–Whitney U test, *p* = 0.232; effect size r = 0.059), indicating negligible practical significance.

To assess the structure of allelic variation in greater detail, the maximum allele frequency per locus was analyzed as a measure of allelic dominance (Figure 3). A tendency toward higher allele dominance was observed in the elderly group at several loci; however, this difference did not reach statistical significance (Mann–Whitney U test, *p* = 0.232). The corresponding effect size was very small (r = 0.059), indicating negligible practical significance. These findings suggest a potential shift toward a more uneven allele distribution in the elderly population; however, given both the lack of statistical significance and the negligible effect size, this observation should be interpreted with caution.

These observations may reflect a tendency toward a more uneven distribution of allele frequencies in the elderly group. However, given the lack of statistical significance, this pattern cannot be interpreted as a definitive shift in genetic structure and should be considered a preliminary observation.

Hardy–Weinberg equilibrium analysis showed that 12 of the 15 autosomal STR loci did not significantly deviate from equilibrium expectations in either study group after Bonferroni correction. Significant deviations remained for D21S11, TH01, and D19S433 in both the young and elderly groups. The complete HWE results, including the unadjusted and Bonferroni-adjusted *p*-values, are presented in Supplementary Table S1.

### 3.3. Exploratory Mapping of STR-Adjacent Genes to Aging-Related KEGG Pathways

**Figure 4:**
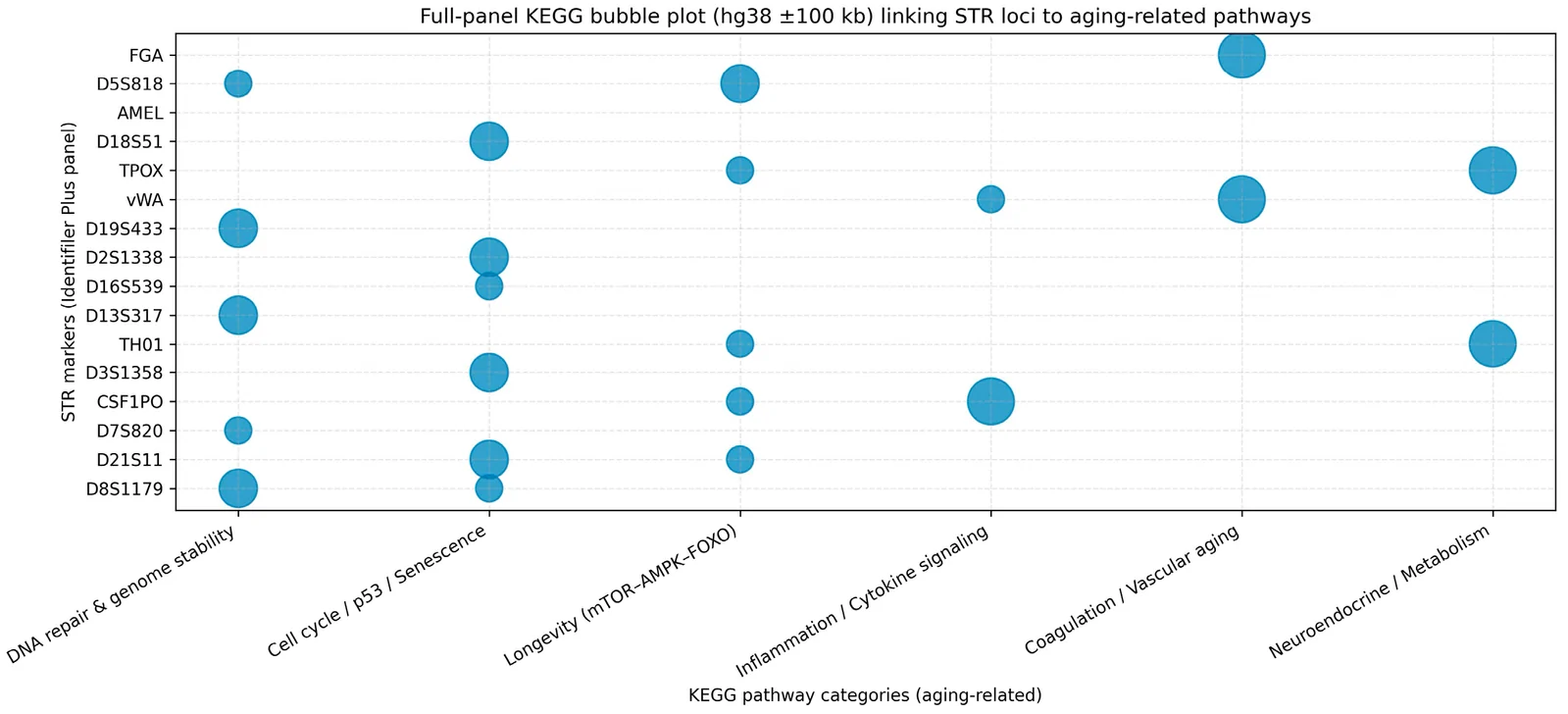
KEGG pathway analysis of genes located within ±100 kb of the investigated STR loci. The analysis is based on genomic proximity and is presented as an exploratory functional annotation intended to provide biological context. The identified pathways should not be interpreted as evidence of direct functional, causal, or statistically enriched biological associations.

To explore the potential biological context of the investigated STR loci, genes located within ±100 kb of each locus in the hg38 reference genome were mapped to KEGG pathway annotations. This procedure was exploratory and did not constitute a formal statistical enrichment analysis.(Figure 4).

This analysis indicated that several STR loci are located in proximity to genes annotated in pathways broadly associated with biological processes relevant to aging, including DNA repair and genomic stability, cellular senescence, inflammatory signaling, longevity regulation, and metabolic processes. However, these findings are based solely on genomic proximity and pathway annotation and do not imply direct functional associations.

## 4. Discussion

The present study suggests a potential trend in STR-based genetic variation between young and elderly populations. However, no statistically significant differences were observed in heterozygosity or allelic dominance between the groups. These findings indicate that while subtle shifts in STR-based parameters may exist, they cannot be interpreted as definitive evidence of age-related genetic divergence. Because the primary comparisons were not statistically significant, the observed patterns should be interpreted as exploratory rather than as evidence of age-related genomic changes. Although these observations may provide a rationale for further investigation, they do not establish a biological or functional relationship between STR-based diversity and aging [9].

Interestingly, although the overall comparison between the young and elderly groups did not reveal statistically significant differences, exploratory subgroup analyses identified differences in germline STR diversity among individuals in the oldest age category. However, these findings reflect interindividual variation between age groups and should not be interpreted as evidence of age-dependent genomic changes occurring within individuals. In particular, individuals aged ≥85 years exhibited lower heterozygosity levels compared with other elderly subgroups and younger individuals. These findings may indicate that STR-based reductions in genetic diversity become more apparent in the oldest-old population rather than across elderly individuals as a whole. However, given the exploratory nature of these analyses and the relatively limited subgroup sizes, these findings should be interpreted cautiously and validated in larger cohorts. In this context, although STR markers do not directly indicate functional effects, they may provide indirect insight into broader patterns of genomic variation. The observed tendencies in heterozygosity and allelic distribution may reflect subtle changes in genomic organization; however, given the lack of statistical significance, these patterns should be interpreted with caution and cannot be considered evidence of true biological differences between the groups [13]. In addition, differences in sample size between the groups may have influenced the distribution patterns and should be considered when interpreting these findings.

**Figure 5:**
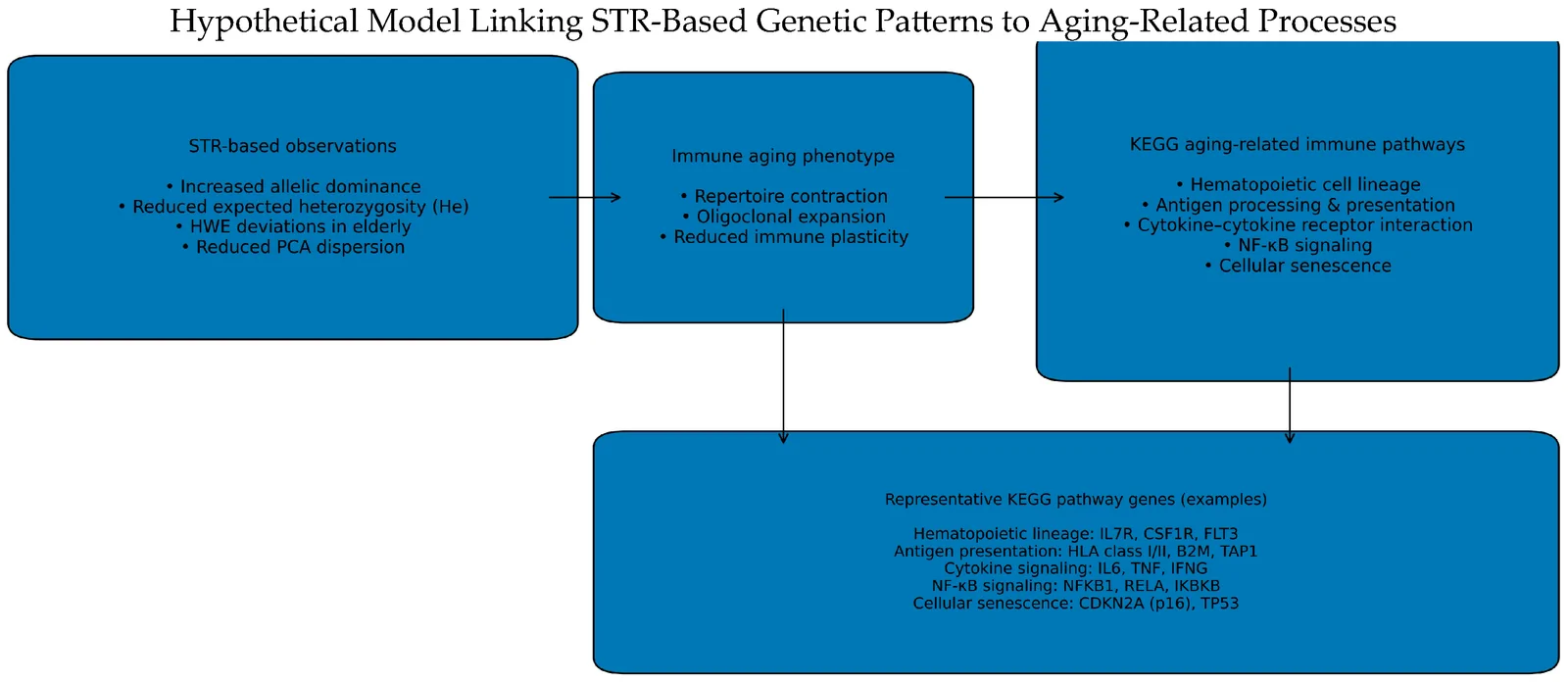
Hypothetical, hypothesis-generating model illustrating potential relationships between STR-based genetic patterns and aging-related biological processes. The proposed relationships are speculative, are based on indirect observations and genomic proximity, and should not be interpreted as experimentally validated functional or causal mechanisms.

The genomic context obtained through KEGG pathway analysis provides a descriptive framework for interpreting the present findings. The proximity of STR loci to genes annotated in pathways broadly associated with aging, including cellular senescence, inflammatory responses, and longevity regulation, may suggest potential biological relevance. However, these observations are based on genomic proximity and pathway annotation and do not imply direct functional, causal, or regulatory relationships. Furthermore, because no predefined background gene set was used, the pathway analysis should be regarded as exploratory and hypothesis-generating. Therefore, these observations require confirmation through future studies employing comprehensive genome-wide enrichment analyses together with complementary functional genomic approaches (Figure 5).

The present findings provide an exploratory basis for future studies investigating whether STR-based variation is associated with age-related genomic patterns. However, the current results do not support the use of STR markers as indicators of biological aging [13]. The study has several limitations. Differences in population structure, sample size, and cohort effects may have influenced the observed results. Furthermore, the largely neutral nature of STR markers limits the ability to draw direct functional inferences. Future studies integrating these findings with functional genomic, epigenetic, and transcriptomic data will be necessary to better understand the biological relevance of the observed patterns [9].

After Bonferroni correction, D21S11, TH01, and D19S433 deviated from Hardy–Weinberg equilibrium in both age groups. Because the same loci showed deviations in both groups, these findings are unlikely to represent an age-specific pattern. They may instead reflect locus-specific characteristics, population substructure, or technical factors. Therefore, locus-specific heterozygosity estimates for these markers should be interpreted with caution.

Additionally, potential confounding factors such as population stratification and cohort effects may have influenced the observed patterns, despite sampling from the same geographical region. The use of a limited set of 15 CODIS STR loci may also restrict the ability to capture genome-wide diversity patterns. Furthermore, technical factors such as allele drop-out during PCR amplification and capillary electrophoresis may have affected heterozygosity estimates, particularly in samples with variable DNA quality.

## 5. Conclusions

In conclusion, the primary analyses did not identify statistically significant differences in STR-based heterozygosity or allelic dominance between the young and elderly groups. Although exploratory subgroup analyses suggested lower individual heterozygosity among the oldest participants, these findings should be interpreted cautiously and require confirmation in larger cohorts. Similarly, the genomic proximity of STR loci to genes annotated in aging-related pathways does not establish direct functional or biological relationships. Collectively, these findings should be regarded as hypothesis-generating and warrant validation in larger, independent prospective cohorts, together with complementary functional genomic studies.

## Data Availability

The data presented in this study are available on reasonable request from the corresponding author. The data are not publicly available due to privacy and ethical restrictions.

## Supplementary Materials

The following supporting information can be downloaded at: https://www.mdpi.com/article/10.3390/genes17080897/s1. Supplementary Table S1: Hardy–Weinberg equilibrium analysis of 15 STR loci.

## Abbreviations

The following abbreviations are used in this manuscript:

STR	Short tandem repeat
MSI	Microsatellite instability
MMR	Mismatch repair
KEGG	Kyoto Encyclopedia of Genes and Genomes
He	Expected heterozygosity
